# Policy challenges for the pediatric rheumatology workforce: Part I. Education and economics

**DOI:** 10.1186/1546-0096-9-24

**Published:** 2011-08-16

**Authors:** Michael Henrickson

**Affiliations:** 1 Division of Rheumatology, MLC 4010, Cincinnati Children's Hospital Medical Center, 3333 Burnet Ave, Cincinnati, OH 45229-3039, USA

## Abstract

For children with rheumatic conditions, the available pediatric rheumatology workforce mitigates their access to care. While the subspecialty experiences steady growth, a critical workforce shortage constrains access. This three-part review proposes both national and international interim policy solutions for the multiple causes of the existing unacceptable shortfall. Part I explores the impact of current educational deficits and economic obstacles which constrain appropriate access to care. Proposed policy solutions follow each identified barrier.

Challenges consequent to obsolete, limited or unavailable exposure to pediatric rheumatology include: absent or inadequate recognition or awareness of rheumatic disease; referral patterns that commonly foster delays in timely diagnosis; and primary care providers' inappropriate or outdated perception of outcomes. Varying models of pediatric rheumatology care delivery consequent to market competition, inadequate reimbursement and uneven institutional support serve as additional barriers to care.

A large proportion of pediatrics residency programs offer pediatric rheumatology rotations. However, a minority of pediatrics residents participate. The current generalist pediatrician workforce has relatively poor musculoskeletal physical examination skills, lacking basic competency in musculoskeletal medicine. To compensate, many primary care providers rely on blood tests, generating referrals that divert scarce resources away from patients who merit accelerated access to care for rheumatic disease. Pediatric rheumatology exposure could be enhanced during residency by providing a mandatory musculoskeletal medicine rotation that includes related musculoskeletal subspecialties. An important step is the progressive improvement of many providers' fixed referral and laboratory testing patterns in lieu of sound physical examination skills.

Changing demographics and persistent reimbursement disparities will require workplace innovation and legislative reform. Reimbursement reform is utterly essential to extending patient access to subspecialty care. In practice settings characterized by a proportion of Medicaid-subsidized patients in excess of the national average (> 41%), institutional support is vital. Accelerating access to care will require the most efficient deployment of existing, limited resources. Practice redesign of such resources can also improve access, e.g., group appointments and an escalating role for physician extenders. Multidisciplinary, team-oriented care and telemedicine have growing evidence basis as solutions to limited access to pediatric rheumatology services.

## Background

A central mission of the pediatric rheumatology (PR) workforce is to provide children with access to care and superior clinical outcomes. This series will examine several educational and economic barriers to workforce development, synthesizing the available data into specific policy goals.

### Challenges

Beyond the known determinants of access to care summarized in Table 1[1-4], PR faces specific challenges. These include three explicit challenges which are consequent to obsolete, limited or unavailable exposure to PR: 1) a) absent or inadequate recognition or awareness of rheumatic disease by primary care providers, patients and their families; b) referral patterns that commonly foster delays in timely diagnosis (e.g., consultation with orthopedic surgeons, neurologists or alternative care practitioners); and c) primary care providers' inappropriate or outdated perception of outcomes. This article will address these three difficulties as a single barrier. Other specific challenges include: 2) varying models of PR care delivery consequent to market competition, inadequate reimbursement and uneven institutional support; 3) compromised quality of care due to current health system delivery, with limited patient access to self-management programs and multidisciplinary team care; and 4) an insufficient workforce supply available to meet the current demand [5]. Substantive improvement in patient access to care will take a coordinated effort to address all of these determinants, in addition to the specific need to increase the number of practicing PR clinicians.

**Table 1:** Determinants of Access to US Pediatric Specialty Care [1-3]

Determinant	Promotes	Obstructs
Income	> 200% federal poverty level (FPL)^‡^	100-200% FPL ("near poor")

Race	White	Minority status or ethnicity

Transportation	Reliable	Inadequate

Parental education	High school diploma or higher	Failure to complete high school

Patient age	2-5 or 13-17 year olds	6-12 year olds

Geographic proximity*	Urban location	Distant location

Medical insurance^Δ^	Present	Absent or under-insured

Cultural or language differences	Absent	Present

^‡ ^Gross annual income for family of 2 at 100% FPL: in the lower 48 states & District of Columbia = $14,710; in Alaska = $18,380; in Hawaii = $16,930 [4].

*Geographic proximity to care does not invariably lead to access.

^Δ ^Although coverage improves the likelihood of access, it is not a guarantee.

Further, each of these challenges bears additional inherent difficulties. Primary care providers may be compromised by deficiencies in 1) musculoskeletal physical examination skills, 2) knowledge of rheumatic disease signs and symptoms, or 3) the knowledge of the importance of early, aggressive treatment. Families may be limited by their capacity to obtain time from work, reliable transportation or child care for the patient's siblings. Several pediatric subspecialties without sufficient workforce to meet demand, including PR, may have lengthy delays in their next available appointment. While the subspecialty's quality improvement initiatives and best practices evolve, current PR care rendered without a multidisciplinary team coordinating care may be inefficient, delaying timely access. Institutional or practice constraints on the development of comprehensive care models which include allied health professionals and self-management programs are commonly attributed to cost, as well as space and time. These interventions are not reimbursed, or remain very limited or unavailable as cost-saving measures. This is a short-sighted strategy, frequently resulting in considerably greater downstream societal and health costs than would be incurred if services had been rendered early in a disease course. In many instances, the consequence of this restricted strategy is also burdensome for the subspecialist, who must function as case manager with marginally available support services.

Many PR practices face escalating access demands during current austerity. This era requires health policies focused on reforming the inadequacies, inequities and inefficiencies of the United States (US) health care system [6]. In 2009, the health share of the US gross domestic product was 17.3%, the largest documented one-year increase (1.1%) since 1960 [7,8]. The current impetus for sustaining health reform stems from a public interest in limiting spending. However, improvement in outcome garners less political and financial attention.

Interim policy solutions need to focus on health promotion through public awareness campaigns (e.g., the Ad Council's launch for the Arthritis Foundation), targeted educational strategies for multiple primary care trainee and practitioner levels, and improvements in PR practice efficiency. Analysis of workforce supply and demand illustrates potential future, long term policy approaches.

Parts I and II of this series will address the four major barriers identified, then offer proposed solutions based on policy analysis of current workforce data. Since trainees' decision-making process is integral to workforce analysis, much of the available data largely derives from studies of general pediatric residents. These studies provide the context for the choices made by aspiring subspecialty trainees, including PRs. Part III will focus specifically on the international workforce needs of PR.

### Barriers & Solutions

#### Barrier 1: Obsolete, Limited or Unavailable Exposure to Pediatric Rheumatology

An essential feature of residency education is to provide training in the management or co-management of problems that are new or unfamiliar. Of 683 general pediatricians surveyed during 2002-06 who were between 1-5 years post-residency training, 74% reported they were rarely or never involved in children requiring PR care [9]. This represented the highest proportion of any subspecialty in the survey. Twenty three percent reported they were sometimes and 3% reported frequently involved in the care of such children. Interestingly, 78% of respondents reported they were comfortable co-managing cases requiring rheumatology care. However, 21% reported being uncomfortable participating in rheumatology care. This proportion was similar to genetics (21%), hematology/oncology (24%) or mental health (20%). Those with local access to subspecialists were more likely to report feeling comfortable managing patients than those without local access. How do the majority of generalists achieve this seemingly incongruent position of being comfortable co-managing children with diseases they rarely or never see? In the era of limits on resident duty hours mandated by the American College of Graduate Medical Education (ACGME), how does the Residency Review Committee (RRC) for Pediatrics ensure an adequate exposure to the spectrum of pediatric subspecialties? Rheumatology remains one of the eleven core electives defined by the RRC for Pediatrics [10]. For rheumatology, the divide between perception and experience may have its origin in residency training.

One-third of programs did not have PR faculty in a 2004 survey of 127 pediatric residency training programs (65% of the total of 195). Nevertheless, these programs successfully involved PRs in their resident training [11]. The survey identified 79% residency programs which offer PR rotations. More than 40% of pediatric residency programs lack an on-site PR. Yet, of the programs offering PR rotations, ≤ 25% of residents participated in the rotation. To succeed in developing the medical home, general pediatricians must be prepared to participate in the care of children with rheumatic diseases and other subspecialty diseases. The choice of most pediatric residents not to participate in this available elective may be the result of perceived irrelevance or competing interests within limited, flexible elective time. Prior surveys of pediatric generalists and subspecialists within their first five years of practice offer insights. Ninety one percent of generalists and 84% of subspecialists would have organized their residency in another way if given 6-12 months of flexible time [9,12]. Freed et al have suggested that pediatric residents may prefer a set, structured curriculum instead of one in which they have responsibility for choosing their educational experiences [13]. A policy approach to reconcile the gap between flawed perception and the need for training in rheumatology is to provide a required musculoskeletal medicine rotation during pediatric residency.

The current generalist pediatrician workforce has relatively poor musculoskeletal physical examination skills. The majority of those currently graduating from U.S. medical schools fail to demonstrate basic competency in musculoskeletal medicine on the physical examination. In a 2004 survey of 100 randomly selected ACGME-accredited residency programs, third-year pediatric residents rated teaching of joint examinations and the pre-participation sports medicine physical as the most poorly taught components of the physical examination. Of the programs surveyed, 29% did not include any specific musculoskeletal or joint examination teaching in their curriculum [14-18]. Formulaic subspecialty referral patterns contribute to delays in appropriate diagnosis and treatment due to conformity or a lack of critical, informed judgment [19]. As a surrogate for competent physical examination skills, many primary care providers rely on blood tests without recognition of their drawbacks. These referral practices further divert scarce resources away from patients with frequently unrecognized rheumatic disease who need accelerated access to care [20,21].

The prevalent, inappropriate use of laboratory testing and imaging results in several negative consequences. These include substantial health care inefficiencies, family and patient anxiety associated with false positive test results, over-reliance on test results instead of physical examination findings of musculoskeletal disease, lost time for patients from school, and lost time from work for their family members [22,23]. In 2006, the American Academy of Pediatrics (AAP) Section on Rheumatology submitted an evidence report development proposal for "Autoantibody Testing in Inflammatory Rheumatic Disease" to the Agency for Healthcare Research and Quality (AHRQ). AHRQ is in the process of completing its draft report following public comment. This report is the second pediatric topic AHRQ prepared for a clinical practice guideline. This evidence-based policy approach hopes to limit currently excessive, misguided autoantibody testing in children and adolescents with non-inflammatory conditions. Such testing adds no additional quality to care or improvement in outcome, while contributing to the rising cost of care. The AHRQ report focuses on clinical guidelines which foster appropriate use of these autoantibody tests.

#### Solution 1: Resident Rotation in Musculoskeletal Medicine

In 2005, the Association of American Medical Colleges embarked on a national reform plan to improve musculoskeletal medicine training in US medical schools [24]. Musculoskeletal complaints are among the most common reasons for children to seek care from their primary care provider [25,26]. However, most practitioners have had little or no clinical training in musculoskeletal health. A 2003 survey indicated that only 47% of US medical schools (57/122) required musculoskeletal medicine training. This finding prompted calls for substantial reform. The American Academy of Orthopaedic Surgeons, the American Medical Association, the Association of American Medical Colleges, the National Board of Medical Examiners, and the US Bone and Joint Decade directed a national effort to promote musculoskeletal medicine education. Follow-up survey results published in 2011 found that 83% of medical schools (106 of 127) now require either a preclinical course (100/127 = 79%) or a clinical rotation (6/127 = 4%) in musculoskeletal medicine [27]. The clinical rotation must involve orthopedics, rheumatology or physiatry. The latter survey did not account for either the duration of instruction or its quality. The task lying ahead will be the hard work of ensuring high quality, sufficient instructional length and sustained curricular prominence for musculoskeletal medicine at medical schools.

There is evidence that musculoskeletal medicine material learned in residency training may be better retained than during medical school training [28]. This supports a policy approach to sustain required musculoskeletal medicine training during residency. The proposed one-month requirement during pediatric residency training could be satisfied by a combined rotation in pediatric orthopedic surgery, sports medicine and rheumatology. Other specialties that could augment the core experience include radiology, genetics (dysmorphology), physical medicine and rehabilitation, and pathology (for the clinical immunology laboratory).

The AAP Section on Rheumatology has twice submitted this proposal to the RRC for Pediatrics (in 2007 and 2009); the RRC has still not responded at the time of this article's completion. Effective policy must address generalists' educational deficit by refocusing on competency-based training, emphasize the need for evidence based clinical guidelines, and contain costs by limiting the use of inappropriate diagnostic testing. These approaches are urgently needed given the increasing limits on resident duty hours.

The American College of Rheumatology (ACR) and the AAP partner to coordinate an annual pharmaceutical industry-endowed, PR Visiting Professor Program [29]. The program sponsors 7-10 professors per year. However, the program lacks sufficient capacity to accommodate sustained pediatrics residency training to any single institution. If the PR portion of the proposed musculoskeletal medicine rotation is not available at a pediatrics residency program or its affiliated institutions, then other practical options are possible. An adult rheumatologist from the community may contribute to the rotation. The AAP's PediaLink musculoskeletal medicine online course is an excellent PR training tool [30]. This unique program combines the perspectives of pediatric orthopedic surgery, PR and sports medicine into physical examination techniques and specific case presentations that use a variety of modalities (video, still photos, diagrams, etc.) to illustrate the course material, arranged by anatomic regions. Telemedicine will likely provide another PR training option as technological advances evolve. Until PR faculty can be established at all pediatric residency programs, these options provide realistic, interim solutions.

#### Barrier 2: Market Competition, Inadequate Reimbursement and Uneven Institutional Support

A prevailing belief is that children are simply small adults [6]. This, of course, ignores an array of issues, e.g., physiologic differences, distinctive disease processes, dissimilar pharmacokinetics and developmental aspects unique to pediatric care. Health insurance cost containment strategies may supersede distinctions between children and adults. This occurs, for example, when insurers promote treatment rendered by specialists trained primarily in adult care and in adult-oriented facilities as an acceptable alternative. Where PR or Medicine-Pediatrics Rheumatology care is available, such a strategy denies pediatric patients access to pediatric subspecialty care. Low public awareness about PR expertise compounds this issue. Further, over 50% of internist rheumatologists (IRs) involved in the care of children have no or minimal exposure to PR during their training [19]. Yet in a 2002 survey, over 80% of IRs involved in the care of children report receiving contact by pediatricians for referrals [31]. Currently, IRs care for ~60% of pediatric patients with rheumatic diseases [32]. On average, patients aged 16 to 17 years represent ~50% of IRs' pediatric patients [33].

For children and adolescents, the Medicaid program provides documented improvement in health care access [34]. On average, 30% of a pediatrician's patients are covered by Medicaid. Medicaid is a means-tested entitlement program for the poor, providing medical and long-term care to an average of 16% of the US population (50 M of 306 M in 2009). In 2009, 20.5% (63 M) of the US population was enrolled in Medicaid for at least one month [35]. Medicaid is administered by each state. Medicaid policy is shared by each state with the federal government, which pays between 50-76% matching funds based on each state's financial capacity as determined by per capita income [36]. Federal administration of Medicaid occurs through the Centers for Medicare and Medicaid Services, including reimbursement policy. Expansion of Medicaid through the 1997 Balanced Budget Act allows coverage for low-income children whose family incomes are not low enough to qualify for Medicaid. This expansion is the State Children's Health Insurance Program (SCHIP). SCHIP-eligible children have family incomes at 100-200% of the federal poverty level (FPL) [4]. Both SCHIP and Medicaid subsidies comprise 8% of the entire federal budget expenditures [37]. Current proposals for alleviating state and federal budget deficits include calls to cut Medicaid funding. State funding reductions for Medicaid would result in the loss of significant federal matching funds. For physicians who care for children, including pediatric subspecialists, Medicaid reimbursement is miserably insufficient. Adequate Medicaid reimbursement is essential to achieve access to care [34]. Low payment, capitation and paperwork concerns all impact Medicaid participation.

**Figure 1:**
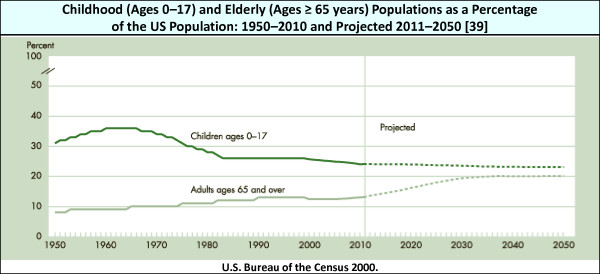
**Comparison of Population Projections: Childhood and Elderly**. The 2000 US Census predicted a 54% population increase for the elderly *vs*. 6.5% for children by 2020.

Reimbursement reform is utterly essential to extending patient access to subspecialty care. Coding does not allow for the distinctive factors affecting the costs of service to a pediatric patient. These factors include the disparity between Medicare and Medicaid reimbursement and uncompensated time spent in the care of children with chronic conditions, e.g., telephone consultations, the need for additional reassurance regarding examinations and other interventions, fear of pain, and the inherent difficulties in communicating directly or effectively with the younger patient [6]. Pediatric billing cannot capture the actual time and energy required to provide quality care. Current payment system inequities exist. These are founded upon 1) relative value units (RVUs) which are used to measure physician productivity, and 2) resource-based relative value scale methodology which is based on the costs of providing adult, not pediatric, care [38]. The former inequity occurs because procedure-based specialties are weighted to higher RVUs than cognitive specialties. In the government subsidy inequity, adult specialists can receive their revenue stream from Medicare, on average providing a third greater reimbursement rates than Medicaid pays to pediatric subspecialists [6]. Among third-party payers, policy analysts and legislators, adult subspecialists maintain substantially greater political and economic influence than pediatric subspecialists based on projected population growth. By 2020, the 2000 US Census predicted population increases for those older than 65 years will increase by 54% *vs*. 6.5% for those 19 years and younger (see Figure 1) [6,39,40].

Along with problems of market competition, inadequate reimbursement, and limited economic influence on reimbursement policy, pediatric subspecialists frequently face limited institutional support. [Personal communication, numerous sources] PRs' reimbursement income and financial influence at their respective institutions are often directly proportionate. Establishing a PR practice at a free-standing children's hospital or a community-based practice is especially daunting because of the requisite outlay of capital with limited direct return on investment. The practice may be unsustainable if attempted in a setting with a particularly high proportion of Medicaid funded patients and income is based solely on direct revenue. This may, in part, influence the choice of at least 72% of PRs who practice at teaching hospitals [41]. Institutions may offset the cost of providing care to high proportions of low-income patients through Medicaid disproportionate share hospital payments. This Medicaid subsidy funds institutions, not providers.

The financial effect of low reimbursement rates for pediatric subspecialists may constrain workforce development in locations that are economically depressed and/or geographically isolated. This can be alleviated by institutional financial support. Such backing may include direct salary subsidies, overhead, and institutional negotiations with managed care medical insurers and grant organizations. Indirect revenue benefiting the institution derives from facility fees, imaging and laboratory services, and allied health professional services, particularly for patients with commercial health insurance.

An institution may potentially lose revenue if imaging, laboratory tests and allied health services are billed to state-subsidized health insurance, e.g., Medicaid or SCHIP. For physicians, the Medicaid-to-Medicare fee index measures each state's physician fees (i.e., payments made to physicians under fee-for-service Medicaid) relative to Medicare fees in each state. Compared to Medicare, only eight states have a Medicaid-to-Medicare fee index above one (AK, AZ, ID, MT, ND, NM, NV and WY; TN does not have a fee-for-service Medicaid program) [42,43]. Most states pay substantially below physician's fees. The national average for the Medicaid-to-Medicare fee index is 0.72. In most states, the PR who provides an increasing volume of visits and services to state-subsidized patients may generate a net loss of revenue to their subsidizing institution. This occurs when reimbursement rates are below cost. During 2003-08, the only change in closing the gap between Medicaid and Medicare reimbursement occurred in primary care and obstetrician fees, when the fee index changed from 0.69 to 0.72. The Health Resources and Services Administration's (HRSA) recommendation to Congress in 2007 concludes: "Increases in the [PR workforce] supply may be accomplished through institutional support for fellowship training, designated salary and research funding for pediatric rheumatologists, and/or improved reimbursement rates." [44]

Comparison of the proportions of total health care spending for the pediatric (0-18 years) and adult (19-65+ years) age groups reveals substantive differences [45]. The health insurance spending ratio of these age groups is equal for both commercial (1.2) and total public to private (1.2) spending. However, the ratio for the two age groups for Medicaid spending is 2.2, underscoring the prominence of Medicaid in lieu of Medicare spending on the pediatric population. As expected, Medicare spending is nearly exclusive to the adult population, with a ratio of 250 for adult/pediatric age groups. The proportion of Medicaid to Medicare reimbursement was 69% in 2003-04 (now 72%). As a national trend, the typical PR would have served over twice the proportion of Medicaid patients as her/his IR counterpart, while receiving 21% lower reimbursement. Twenty eight percent of all health care services for the pediatric age group were for physician and clinical services. However, only 15% of total health care spending in this age group reimbursed physician and clinical services.

It is in this economic environment that the PR must operate. In practice settings characterized by a proportion of Medicaid-subsidized patients in excess of the national average (> 41%), institutional support is virtually a necessity. Market competition among institutions for privately insured patients which improve the overall proportion of service reimbursement can be fervent. This can lead to nonsensical distribution of limited workforce assets. Among competing health care oligopolies, PRs may be diverted through "outreach" to geographic locations currently served by existing PRs. The primarily fee-for-service design of the US health care system responds to market forces, and secondarily to the needs of patient access to care. When this system values access to care as its chief priority, PR workforce distribution may align equitably. In the meantime, PRs will struggle to reconcile the economics of their practice productivity reports, perceived cost-effectiveness to their institution, and the payer mix of their patient population.

Varying models of PR care delivery arise within this economic construct. Considerable variability exists among programs with services for children with special health care needs and their families [46]. Such differences in health care delivery can lead to differences in outcome as well as disparities in care. For example, state-subsidized services for patients with pediatric rheumatic disease vary widely among states. In the US, health care costs contribute to limiting access to care [47]. Medicaid status has been associated with significantly lower health-related quality of life and higher disability in juvenile rheumatoid arthritis compared to commercial medical insurance status [48]. The key drivers and barriers to limited access to care are multi-factorial. Health care financing will continue to be a major feature of care delivery in the US.

Multidisciplinary, team-oriented treatment avoids the negative economic consequences of suboptimal disease treatment (including an enormous, societal financial burden), the patient's early retirement in adulthood from disability, and the patient's lack of integration into society. Further, this approach has been proven to prevent irreparable damage and long-term disability in pediatric rheumatic disease. Cost savings have been demonstrated in both the medium and long-term using the multidisciplinary, team-oriented care practice [49,50]. Specifically, this has been demonstrated for the care of patients with juvenile idiopathic arthritis (JIA) [51-53]. In a study that included analysis of specific JIA characteristics' influence on various costs domains, function was the only factor which significantly contributed to the variation in patient total costs [54]. Randomized clinical trials of multifaceted interventions provided in a multidisciplinary care setting for adult patients with osteoarthritis are currently under study for cost-effectiveness. These will be completed in 2011 [55,56]. JIA serves as the prototypic example in the care of patients with pediatric rheumatic disease. Cost-effectiveness studies will need to establish that multidisciplinary team care should be adopted as the standard in other pediatric rheumatic diseases. Since pediatric rheumatology centers provide care to patients with a variety of these diseases, it is ethically difficult to separate multidisciplinary team care from a restricted/"regular" care model.

#### Solution 2: Reimbursement and Practice Redesign

For now, policy must balance the potential impact of competition with the anticipated limits on PR workforce growth in the foreseeable future. The HRSA report that internist rheumatologists (IRs) fill the PR shortfall has pragmatic merit [44]. Policy advances will occur by researching the extent to which IRs can provide quality pediatric care [57]. In the interim, primary care specialists and physician extenders practicing in underserved areas without PR need pediatric musculoskeletal medicine training. A mechanism available for this is HRSA's Area Health Education Centers program. The collective goal is to provide patients with timely access to PR care, per Congressional authorization [58].

All states should regularly review Medicaid reimbursement rates and increase them at least to parity with Medicare. Changes promoting equitable compensation will require substantial revision of the entire payment scheme through US health care system reform. Health care reform was a major focus of the Obama administration during 2009-10. Sustaining landmark progress in reform is an ongoing effort of this administration. Recent financial and health reform legislation initiatives included federal stimulus funds (American Recovery and Reinvestment Act of 2009), ongoing temporary *vs*. permanent change in the sustainable growth rate (a statutory formula based on the SGR determines Medicare physician compensation) and Medicaid spending cuts despite a recent 7.5% increase in total enrollment (3.3 million people during 2008-09, including 2 million children) [59-61]. Total Medicaid enrollment increased during 2009 by the largest amount since the early days of program implementation in the late-1960s [61]. Projected enrollment for 2009-10 will continue to rise with a 5.6% increase.

While these reforms ensue, one policy strategy is the development and propagation of telemedicine as a modality which can provide access for patients in remote locations. A non-inferiority, randomized clinical trial of adult patients compared telemedicine to in-person consultations at pulmonary, endocrine and rheumatology clinics. Patient satisfaction with physician's clinical competence, interpersonal skills (including development of rapport), use of shared decision-making, and promotion of patient-centered communication was similar. Patients reported greater satisfaction with convenience for telemedicine compared to in-person consultations [62]. Telemedicine is cost-effective as an alternative care delivery model for patients needing adult rheumatology services that reside in remote rural and northern communities in Labrador, Canada [63]. Cost-benefit analysis has been inconclusive to date due to the relative paucity of comparable data [64]. Long-term outcome data are also lacking [65]. An observational prospective study of telemedicine involving IR consultations provided in Belfast, UK indicated a diagnostic accuracy of 97% based on follow-up, comparison, in-person consultations [66]. While there is no published experience of telemedicine-based PR consultation, this care delivery model holds promise for patients in remote locations. Certainly, this strategy merits investigation as a means of extending available PR workforce and improving access to care.

PR practices should consider a number of redesign strategies to improve efficiency and the need to maximize limited resources. Appointment failures ("no-shows") can be improved by decreasing appointment lead time to no greater than a 2-3 month interval; beyond this, the failure rate approaches 35% [67,68]. Open-access scheduling (same day appointments), used by primary care practices, virtually eliminates appointment failures due to far-in-the-future, forgotten appointments or the discovery of another consultant. Partial open-access or a "fast track" clinic may be successful strategies for PR practice scheduling. Group appointments for patient and family support, education and self-management skills provide a welcome opportunity for families to network with each other and allow practices to deploy their resources efficiently. Genetics, obesity and diabetes management clinics offer precedent. A family-centered parent council could facilitate such opportunities. Fundamental reimbursement reform will be important for the success of these strategies.

During advance review of scheduled patients, common symptom clusters allow allied health staff to identify patients who will likely need their services. Nurses can provide case management of complex patients. Physician extenders can provide routine care, allowing PRs to consult on complex cases.

PR needs to develop consensus about its role in the ongoing management of patients with chronic widespread pain disorders, who comprise up to 25% of new patients [69-72]. In many underserved geographic locations, pain management services can be provided through a variety of collaborative community resources.

### Summary of Policy Recommendations

Expansion of the PR workforce will require a multi-pronged approach that addresses several unique challenges. Existing barriers especially relevant to PR entail a broad scope of inadequate awareness about pediatric rheumatic disease involving primary care providers, families and patients. Strategies to improve primary care providers' education, together with physician extenders, include competency-based training, evidence based clinical guidelines, and cost containment limiting the use of inappropriate diagnostic testing. The AAP's PediaLink musculoskeletal medicine online course, reconfiguration of available residency program assets (e.g., other musculoskeletal subspecialties), and local IRs serve as existing options for programs that lack a PR on faculty. In some locations, PRs are actually proximate to residency programs lacking a PR on faculty. Politics, regional medical insurance plan limits on the number of approved providers, and institutional bureaucracies need to be overcome to allow residents access to essential PR training and clinical experience. The ACR/AAP PR visiting professor program is a 1-2 day, highly focused option which is especially well-suited to geographically underserved locations. Telemedicine holds much potential for education, in addition to consultation. IRs may provide an interim role for juvenile rheumatic disease care delivery, although many have quite limited training in PR. The growing cadre of rheumatologists dually trained in Internal Medicine and Pediatrics has potential for alleviating the shortfall in patient access to PR care.

Nationally, viable policy solutions include:

1. Mandatory musculoskeletal medicine competency-based training during the first two years of residency

2. Limiting low-risk referrals with positive autoantibody testing, using federal quality of care directives

3. Increasing physician extenders' musculoskeletal medicine training and scope of practice

4. Initiating reimbursement reform that brings parity to Medicaid and Medicare payments, and that acknowledges and compensates providers for the distinctive factors affecting the costs of service to pediatric patients with chronic conditions

5. Increasing technological capacity and infrastructure to provide access for geographically isolated patients in underserved regions via telemedicine

6. Redesigning practices to consolidate and deploy services efficiently

7. Sustained health promotions initiatives to increase public awareness about rheumatic diseases.

## Conclusions

Improving patient access to care is the principal objective of alleviating the dearth of available PR workforce. Existing resources can be thoughtfully reorganized to provide musculoskeletal medicine training. This is preferable to an absence of musculoskeletal education. Innovative programs exist to provide such instruction. National health care quality guidelines will shortly be available addressing the appropriate use of laboratory testing in pediatric musculoskeletal conditions. Reimbursement reform is an essential element to improving pediatric subspecialty care access. The next article in this series will explore how reimbursement patterns and a health care delivery system in the US oriented to acute medical care rather than chronic condition management interact to exacerbate PR workforce challenges. Trainees' career choices are shaped by a variety of influences. The role of these financing issues serves as another important factor in understanding how to accelerate resolution of the PR workforce deficit. In the meantime, PR practices can improve their efficiency and maximize resources through several creative strategies, such as partial open-access scheduling, group appointments, limitations on lengthy follow-up intervals in the appointment schedule, and expansion of the role of physician extenders who develop expertise in musculoskeletal medicine.

## Abbreviations

AAP: American Academy of Pediatrics; ACGME: American College of Graduate Medical Education; ACR: American College of Rheumatology; AHRQ: Agency for Healthcare Research and Quality; FPL: federal poverty limit; HRSA: Health Resources and Services Administration; IR: internist rheumatology/rheumatologist; JIA: juvenile idiopathic arthritis; PR: pediatric rheumatology/rheumatologist; RRC: Residency Review Committee; US: United States.

## Competing interests

Dr. Henrickson is a current member of the ACR Committee on Government Affairs. He has no competing financial interests to disclose. The content of this article does not reflect any official position or policy of the ACR.

## Authors' contributions

MH contributed all aspects of this article.
